# Central nervous system vasculopathy caused by Fabry disease: a case report

**DOI:** 10.1186/s12883-019-1348-9

**Published:** 2019-06-06

**Authors:** De-Zheng Kong, Ya-Hui Lian, Lin-Jing Wang, Chun-Mei Wang, Yang-Yang Meng, Hong-Wei Zhou

**Affiliations:** Department of Radiology, The First Hospital of Jilin University, Xinmin St. #71, Changchun, 130021 China

**Keywords:** Fabry disease, Vasculopathy, Central nervous system

## Abstract

**Background:**

Fabry disease is rare, and the diagnosis is often delayed. Here, we describe a case of Fabry disease resulting in vasculopathy of the central nervous system. Magnetic resonance (MR) black-blood sequence (three-dimensional T1 volumetric isotropic turbo spin echo acquisition), with the unique advantage of imaging the vascular wall, facilitated a clear identification of the vasculopathy.

**Case presentation:**

A 27-year-old man visited our hospital for the treatment of “ double vision 6d.” After a series of examinations, the patient was diagnosed with Fabry disease, which caused vasculopathy of the central nervous system. Subsequently, the patient was treated with corticosteroids and his symptoms were attenuated. Two months after the initial treatment, the initial lesion in the vascular vessel disappeared, however, a new lesion appeared. Similarly, four months after the initial treatment, although the previous lesion disappeared, a new lesion appeared.

**Conclusions:**

This case highlights that clinicians should use MR black-blood sequence scan in a timely manner in case of young patients with migratory lesions of brain. In case of detection of a vascular lesion in combination with other systemic lesions, the possibility of Fabry disease should be considered.

## Background

Fabry disease is an X-linked inherited α-galactosidase deficiency disease. The disease is rare and more likely to occur in males, mostly during childhood and adolescence. The incidence rate varies approximately between 1/476000 and 1/117000 [1, 2], and the diagnosis of the disease is often delayed. The average time interval between symptom onset and diagnosis is 12.5 years for male patients with Fabry disease, while that of female patients is 13.1 years [3].

## Case presentation

**Fig. 1:**
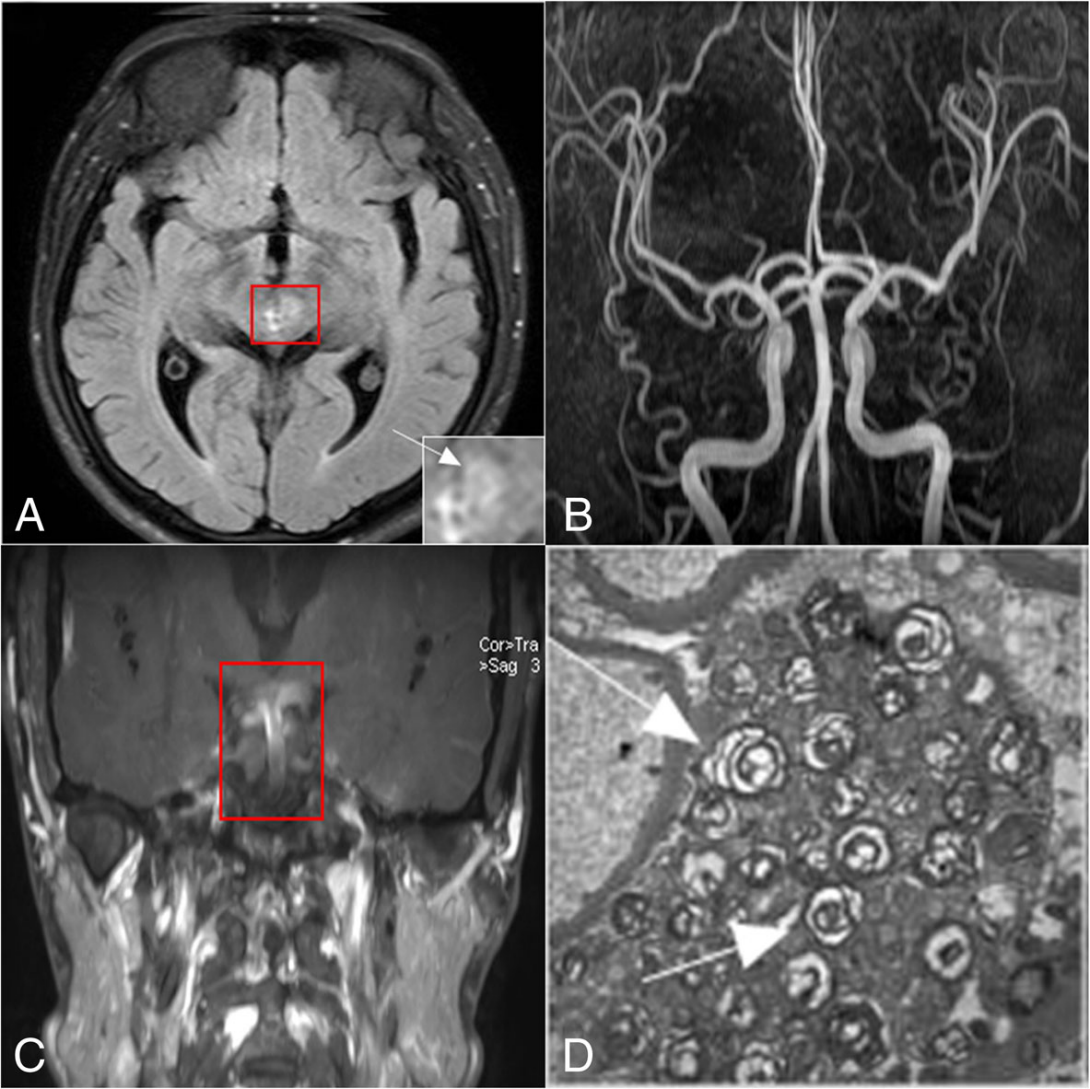
**a**: MR-FLAIR image of the brain with abnormal signals in the left oculomotor nucleus. **b**: No obvious abnormality in 3D-TOF-MRA. **c**: The black-blood sequence image with partial thickening and mild enhancement of the basilar artery and bilateral posterior cerebral artery. **d**: Kidney pathological biopsy: a large number of myeloid bodies and zebra bodies (arrow mark)

**Fig. 2:**
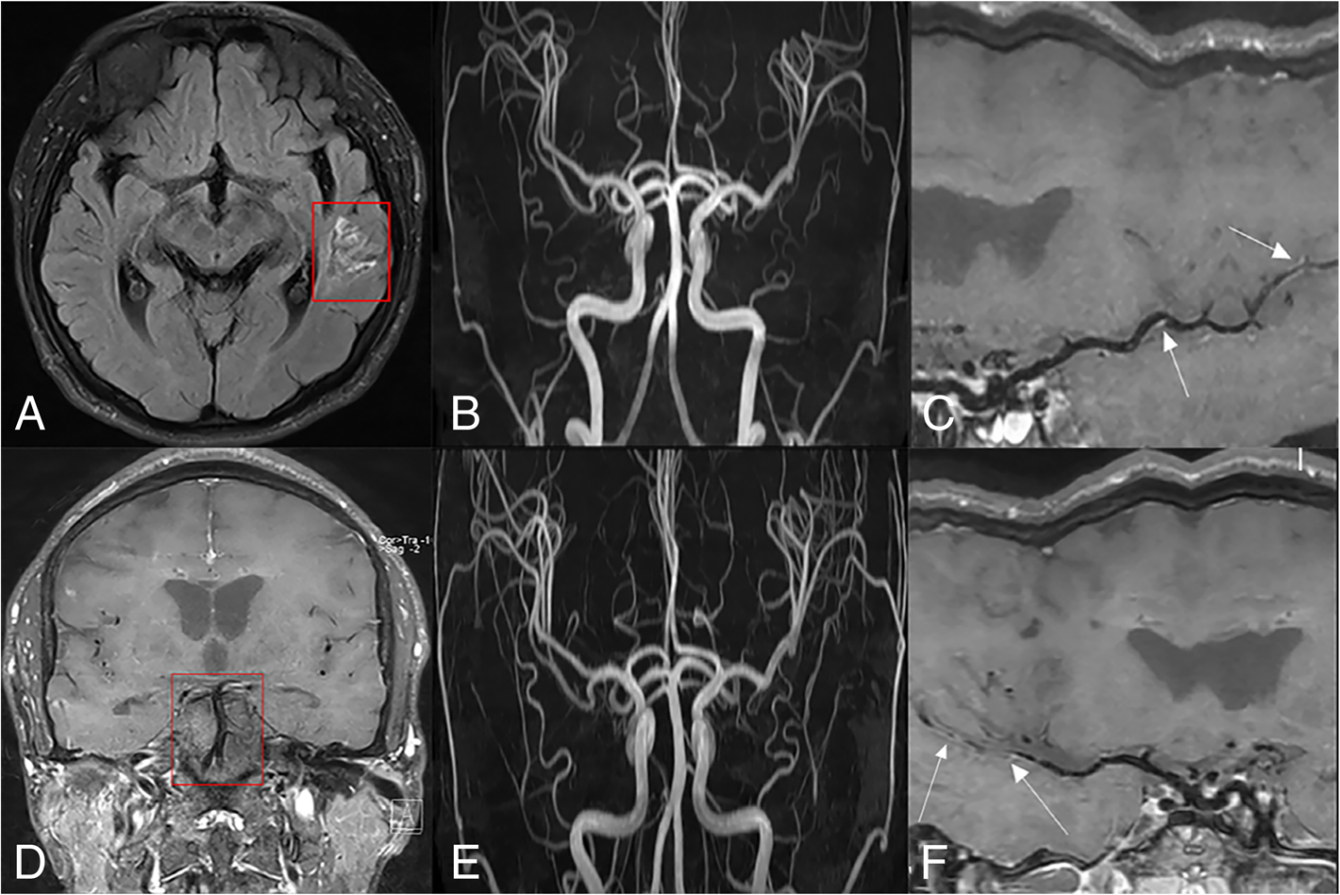
**a**: MR-FLAIR image of the brain, two months later after initial treatment, with new abnormal signals in the left temporal lobe. **b**: No obvious abnormality in 3D-TOF-MRA. **c**: The black-blood sequence image with an abnormal enhancement of the left middle cerebral artery (arrow mark). **d**: The black-blood sequence image detecting that abnormal enhancement of the basilar artery and bilateral posterior cerebral artery had disappeared. **e**: No obvious abnormality in 3D-TOF-MRA four months after initial treatment. **f**: The black-blood sequence image with local thickness and abnormal enhancement of the right middle cerebral artery four months after the initial treatment (arrow mark)

A 27-year-old man was admitted to the hospital owing to “double vision 6d.” His admission examination results revealed absence of left eye adduction, and he had an outward squint. His auxiliary examination results were as follows: proteinuria: 3+, urinary occult blood: 1+, 24-h proteinuria: 3.44 g/24 h (normal: < 0.20 g/24 h); cerebrospinal fluid pressure: 240 mmHg, glucose: 2.05 mmol/L, white blood cell count: 23 × 10^6^/L, protein: 0.67 g/L, and cerebrospinal fluid immunoglobulin IgG: 68.00 mg/L (normal: 0–34.0 mg/L); activity of α-galactosidase A: 0.9 nmol/(h.mg) (normal: 25.5~64.1 nmol/(h.mg)). MR-fluid attenuated inversion recovery (FLAIR) imaging of the brain showed abnormal signals in the left oculomotor nucleus (Fig. 1a), and no obvious abnormality was noted on three-dimensional time-of-flight magnetic resonance angiography (3D-TOF-MRA) (Fig. 1b). The black-blood sequence showed partial thickening and mild enhancement of basilar artery and bilateral posterior cerebral artery (Fig. 1c). A pathological biopsy of the kidney revealed a large number of myeloid bodies and zebra bodies (Fig. 1d). Genetic testing revealed a nucleotide mutation in the GLA gene c.426C > A (nucleotide in the coding region 426 from C to A), which caused the Cys 142 amino acid-translating codon to function as a stop codon (p. Cys142Ter); this phenomenon led to the early termination of peptide chain synthesis. The mutation has been reported in the literature to be associated with Fabry disease (reference databases HGMD Pro and PubMed) [4]. The frequency of this mutation in the population is extremely low (reference databases: 1000 Genomes, dbSNP). According to the abovementioned test results, the diagnosis was Fabry disease. Subsequently, we treated our patient with corticosteroids, and he was discharged after his symptoms ameliorated. The patient was treated with long-term aspirin after discharge from the hospital. The patient returned to our observation after 2 months; MR-FLAIR imaging of the patient’s brain revealed new abnormal signals in the left temporal lobe (Fig. 2a), but we could not detect any obvious abnormality on the 3D-TOF-MRA (Fig. 2b). Furthermore, the black-blood sequence revealed an abnormal enhancement of the left middle cerebral artery (Fig. 2c), and the abnormal enhancement of the basilar artery and bilateral posterior cerebral artery had disappeared (Fig. 2d). Four months later after initial treatment, the black-blood sequence revealed that the abnormal enhancement of the left middle cerebral artery had disappeared, and no obvious abnormality could be detected in the 3D-TOF-MRA (Fig. 2e); however, local thickness and abnormal enhancement of the right middle cerebral artery was detected in the black-blood sequence (Fig. 2f). There were no symptoms during follow-up.

## Discussion and conclusions

Fabry disease is caused by a mutation or deletion of a gene on the chromosome Xq22 that causes partial or total deficiency of α-galactosidase A, which impairs catabolism of trimeric hexose ceramide, which progressively accumulates in the kidney, heart, vascular wall, and the nervous system. Accumulation in tissue cells causes subsequent damage to multiple organ systems [5]. In the later stages of the disease, multiple organs such as the kidney, heart, and other organs are progressively damaged. Most patients reportedly die of uremia or cardiovascular complications at 40–50 years of age [6]. As in our patient, recurrent strokes, causing lesions in different vascular distribution area, have a huge negative impact on the patient’s quality of life. Therefore, timely and accurate diagnosis is crucial for patients. However, as mentioned above, the disease often has a delayed diagnosis, which is unfavorable for disease prognosis. Therefore, in case of repeated strokes in young patients and the occurrence of migratory lesions without any obvious abnormalities noted during the 3D-TOF-MRA examination, the MR black-blood sequence scan should be used to check for vasculopathy. If vasculopathy exists accompanied by other systemic damage, the possibility of Fabry disease should be considered. Renal biopsy and genetic testing should be subsequently performed to confirm this. The treatment of the disease includes both non-specific and specific approaches. The non-specific approach involves symptomatic treatment, whereas the specific approach involves enzyme replacement therapy. Our patient could not afford the expensive enzyme replacement therapy. Studies have shown that corticosteroid therapy can result in short-term efficacy in patients with vascular lesions that affect the central nervous system [7]. Therefore, we used corticosteroid therapy for our patient and achieved beneficial effects. According to the patient’s condition, we used intravenous methylprednisolone drip, starting at 80 mg/d for the initial 5 days, and then oral prednisone at 60 mg/d (1 mg/kg/d) with dose reduction of 20 mg/3 d until withdrawal. He was discharged after his symptoms improved, and then, he was treated with long-term aspirin**.**

## Data Availability

All data related to this case report are documented within this manuscript.

## Abbreviations

3D-TOF-MRA: three-dimensional time-of-flight magnetic resonance angiography
FLAIR: fluid attenuated inversion recovery
MR: magnetic resonance
